# Association between obesity and asthma among teenagers

**DOI:** 10.1590/S1516-31802008000500008

**Published:** 2008-09-04

**Authors:** Maria do Pilar Carneiro Bertolace, Eliana Toledo, Patrícia Polis de Oliveira Jorge, Raphael Del Roio Liberatore

**Keywords:** Asthma, Body weight, Obesity, Body mass index, Adolescent, Asma, Peso corporal, Obesidade, Índice de massa corporal, Adolescentes

## Abstract

**CONTEXT AND OBJECTIVE:**

Obesity and asthma are serious and growing problems. Since adipose tissue produces inflammatory substances, the aim of this study was to estimate the prevalence of asthma among students at schools in São José do Rio Preto (Phase 1), and to corroborate the hypothesis for an association between obesity and asthma among these students (Phase 2).

**DESIGN AND SETTING:**

Cross-sectional study at Faculdade de Medicina de São José do Rio Preto (Famerp).

**METHODS:**

The study consisted of two successive and dependent stages. Phase I was a cross-sectional study on 4103 randomly selected students (13-14 years old), to determine the prevalence and severity of asthma. Phase II was an analytical cross-sectional study on 431 students (190 asthmatics and 231 non-asthmatics) from Phase I, to evaluate the hypothesis of an association between obesity measured by the body mass index (BMI) and asthma. To diagnose asthma and obesity, the criteria of the International Study of Asthma and Allergies in Childhood (ISAAC) and the chart from the Centers for Disease Control (CDC; 2000) were used. The data were analyzed using Student's t test.

**RESULTS:**

We found that 5.6% of the students analyzed in Phase I were asthmatic. The BMI among the asthmatic students (21.84 kg/m^2^) was higher than the BMI among the non-asthmatics (21.73 kg/m^2^), although the p value was 0.766.

**CONCLUSION:**

In our study group, we did not find any association between increased BMI and the prevalence of asthma.

## RESUMO

**CONTEXTO AND OBJETIVO:**

Obesidade e asma são sérios e crescentes problemas em nossa sociedade. Como o tecido adiposo produz substâncias inflamatórias, o objetivo foi estimar a prevalência de asma entre os estudantes (Fase 1), e verificar uma hipótese de associação entre obesidade e asma (Fase 2), em escolares de São José do Rio Preto, São Paulo.

**TIPO DE ESTUDO E LOCAL:**

Estudo transversal realizado pela Faculdade de Medicina de São José do Rio Preto (Famerp), São Paulo.

**MÉTODOS:**

O estudo foi realizado em duas etapas sucessivas e dependentes. A Fase I, em estudo transversal de prevalência com 4.103 escolares (13-14 anos de idade), para determinar a prevalência e a gravidade da asma. A Fase II, transversal analítico, com 431 (190 asmáticos e 231 não asmáticos), provenientes do grupo da Fase I, avaliou a associação entre obesidade, classificada por índice de massa corporal (IMC) e asma. As amostras foram selecionadas aleatoriamente. Para os diagnósticos de asma e obesidade foram empregados os critérios do International Study of Asthma and Allergies in Childhood (ISAAC) e a tabela do Centers for Disease Control (CDC) 2000. Os dados obtidos foram analisados utilizando o teste t de Student.

**RESULTADOS:**

Notou-se que 5,6% do grupo analisado na Fase I são asmáticos; e observou-se um aumento do IMC de asmáticos (21,8 kg/m^2^) em relação ao dos não-asmáticos (21,73 kg/m^2^). No entanto, o valor de p obtido foi de 0,766.

**CONCLUSÃO:**

Neste estudo não foi encontrada uma associação entre IMC elevado e prevalência de asma, no grupo estudado.

## INTRODUCTION

Obesity is one of the biggest health problems.^1-3^ It causes great damage, particularly when acquired during childhood.^2,4^ Moreover, fatty tissue is believed to produce inflammatory substances like leptin and interferon, which could hypothetically justify the claim that obesity is a risk factor for allergic illnesses.^5^

Recent studies have attempted to demonstrate an association between obesity and asthma, which is a highly prevalent chronic inflammatory illness (7.2% of the world's population are asthmatic).^6-10^ Some studies have demonstrated that reductions in body mass index (BMI) among patients with asthma improve their pulmonary function, asthma symptoms, morbidity and state of health.^7^ BMI has been found to be a significant prognostic factor for atopy and allergic symptoms among teenage girls.^8,9^

## OBJECTIVE

We proposed to study and estimate the prevalence of asthma among a group of students in São José do Rio Preto, and to create a hypothesis for an association between obesity and asthma.

## METHODS

The study was designed in two successive and dependent stages.

Phase I was conducted in 2003, and consisted of a cross-sectional study to determine the prevalence and severity of asthma among teenagers. All students who at that time were between 13 and 14 years old, from all schools in São José do Rio Preto, were invited to take part. However, some students did not want to take part or did not go to school on the day when the questionnaire was applied. Thus, 4103 adolescents answered the International Study of Asthma and Allergies in Childhood (ISAAC) questionnaire. This questionnaire is designed to search for early symptoms of asthma among the population, and it contains eight different questions about the subjects’ previous history of coughing and wheezing. For individuals to be considered asthmatic, they need to answer "Yes" to at least one of the questions 1, 2, 6, 7 and 8.^11^

Between March and December 2005, we conducted Phase II. We attempted to test for any association between obesity and asthma by means of an analytical cross-sectional study. Hence, all the asthmatics (5.6% of the group analyzed in Phase 1) took part in phase II of the study, together with a similar number of non-asthmatic students. The latter were selected independently of race, gender, social position or location of residence in the city of São José do Rio Preto. Finally, out of the original 4103 adolescents in the study, 431 students were selected randomly. At that time, they were between 15 and 16 years old.

All the students’ weights and heights were measured. The BMI (kg/m^2^) was calculated and plotted on the chart of BMI for age and gender from the Centers for Disease Control and Prevention (CDC; 2000). Diagnoses of overweight and obesity were established when the BMI was higher than the 85^th^ percentile in the chart.^12^

Among these 431 students, 10 were excluded because their BMI was lower than the fifth percentile. Hence, among the remaining 421 students, 231 were considered asthmatic and 190 non-asthmatic, in accordance with the criteria described above.

This study was approved by the Ethics Committee of the Faculdade de Medicina de São José do Rio Preto.

The data were analyzed using Student's t test, to compare differences in mean BMI between the asthmatic and non-asthmatic students.

## RESULTS

In Phase I, we observed that the prevalence of asthma in this age group was 5.6%.

In Phase II, we analyzed 421 teenagers of mean age 15 years: 281 females (66.8%) and 140 males (33.2%). For the whole group, the mean weight was 59.9 kg (standard deviation, SD = 12.1) and the mean height was 1.65 m (SD = 0.07), and thus the mean BMI was 21.8 kg/m^2^ (SD = 3.6).

Among these 421 students, 231 (54.9%) were considered asthmatic, with a mean age of 15.4 years (155 females and 76 males), and 190 (45.1%) were considered non-asthmatic, with a mean age of 15.4 years (126 females and 64 males).

The asthmatic students had a mean weight of 60.2 kg (SD = 12.2), mean height of 1.65 m (SD = 0.08) and mean BMI of 21.8 kg/m^2^ (SD = 3.7). On the CDC chart, 77.0% were classified as eutrophic, 13.4% overweight and 9.6% obese.

The non-asthmatic group had a mean weight of 59.6 kg (SD = 12.1), mean height of 1.65 m (SD = 0.07) and mean BMI of 21.7 kg/m^2^ (SD = 3.6). On the CDC chart, 81.0% were eutrophic, 11.0% overweight and 7.9% obese.

The statistical "p" values are presented in Table 1 and the clinical data in Table 2.

**Table 1.** Differences observed between asthmatic and non-asthmatic students in São José do Rio Preto, in 2005

	Group (n)	Age (years)	Weight (kg)	Height (m)	BMI (kg/m^2^)	Gender Male/Female
**Asthmatic**	231	15.4	60.2	1.65	21.8	76/155
**Non-asthmatic**	190	15.4	59.6	1.65	21.7	64/126
**p-value**	-	0.304	0.604	0.495	0.766	0.865

BMI = body mass index

**Table 2.** Distribution of nutritional status (eutrophic, overweight or obese) among asthmatic and non-asthmatic student, according to gender, in São José do Rio Preto in 2005

	Asthmatic students			Non-asthmatic students			Total		
**Sample**	**Female**	**Male**	**Total**	**Female**	**Male**	**Total**	**Female**	**Male**	**Total**
**Eutrophic**	123	55	178	107	47	154	230	102	332
	(69.1%)	(30.9%)	(77.0%)	(69.5%)	(30.5%)	(81%)	(69.3%)	(30.7%)	(78.9%)
**Overweight**	21	10	31	13	8	21	34	18	52
	(67.7%)	(32.3%)	(13.4%)	(61.9%)	(38.1%)	(11%)	(65.4%)	(34.6%)	(12.3%)
**Obese**	11	11	22	6	9	15	17	20	37
	(50%)	(50%)	(9.6%)	(40%)	(60%)	(7.9%)	(45.9%)	(54.1%)	(8.8%)
**Total**	**155**	**76**	**231**	**126**	**64**	**190**	**281**	**140**	**421**
	**(67.1%)**	**(32.9%)**	**(100%)**	**(66.3%)**	**(33.7%)**	**(100%)**	**(66.7%)**	**(33.3%)**	**(100%)**

## DISCUSSION

Obesity is a worldwide epidemic health problem, even in developing countries. The prevalence of obesity is increasing, caused by different factors like modernization, which stimulates sedentary lifestyles, and the consumption of diets rich in proteins, fats, sugars and salt, as well as genetic factors.^2^

Asthma is another highly prevalent illness that is responsible for high rates of social and economic damage. It is defined as chronic inflammatory illness of the airways. Although the pathogenetic and therapeutic mechanisms for asthma are relatively well known, mortality due to asthma has been increasing since the 1980s all over the world. Its worldwide prevalence is 7.2%, and the prevalence observed in the study population (5.6%), using the methods described above, came close to this number.^7,10,13^

With increasing prevalence of both obesity and asthma, a possible relationship between them has been hypothesized. The existence of possible genetic, biological and physical mechanisms (like gastroesophageal reflux) or chemical mechanisms (inflammatory substances like tumor necrosis factor alpha, leptin or adiponectin) that are common to asthma and obesity has been observed. Based on this, studies carried out all over the world have demonstrated a positive relationship between the two diseases.^14-19^

Studies carried out in Brazil, using similar but younger samples of patients, concluded that increased BMI was not associated with the prevalence and severity of asthma among adolescents, but did find an association with increased prevalence of wheezing.^10,14^

A Canadian study also did not found any positive association between obesity and asthma.^9^ An Australian survey suggested that high BMI was related to increased frequency of coughing and shortness of breath, but not to atopy, hyperresponsivity or aerial blockage.^17^ In two other studies, no association between these illnesses was found.^18,19^

In our study, we were unable to find any significant positive association between increased BMI and the prevalence of asthma.

This discrepancy between hypotheses and results could be due to the complicated diagnosis of asthma. The symptoms and clinical history seem to be insufficient to differentiate between dyspnea and wheezing caused by mechanical or inflammatory factors. Another possible factor could be the number of participants in these studies. Thus, other studies enrolling more subjects, of different ages, might be necessary.

Moreover, with a view to confirming whether there was any association between obesity and asthma, "hypothesis tests" can be used, among them the chi-squared test in studies like cohort studies, which is a statistical test on the fit of associations between variables that makes it possible to analyze whether the behavior of one variable depends on another. In this research, we used Student's test, more appropriate for frequencies.

## CONCLUSIONS

In this study, we did not find any significant positive association between increased BMI and the prevalence of asthma.
